# Interpreting inter- and intra-annual environmental signals in tree-ring **δ**^18^O using isotope-enabled modeling

**DOI:** 10.1093/treephys/tpag026

**Published:** 2026-02-20

**Authors:** Kersti Leppä, Paul Szejner, Charlotte Angove, Giles H F Young, Olga Blokhina, Kurt Fagerstedt, Ansgar Kahmen, Samuli Launiainen, Lan Mo, Daniel B Nelson, Andreas Richter, Elina Sahlstedt, Pauliina Schiestl-Aalto, Yu Tang, Katja T Rinne-Garmston

**Affiliations:** Ecosystems and Modelling, Bioeconomy and Environment, Natural Resources Institute Finland (Luke), Latokartanonkaari 9, FI-00790 Helsinki, Finland; Ecosystems and Modelling, Bioeconomy and Environment, Natural Resources Institute Finland (Luke), Latokartanonkaari 9, FI-00790 Helsinki, Finland; Stable Isotope Laboratory of Luke (SILL), Natural Resources Institute Finland (Luke), Latokartanonkaari 9, FI-00790 Helsinki, Finland; Stable Isotope Laboratory of Luke (SILL), Natural Resources Institute Finland (Luke), Latokartanonkaari 9, FI-00790 Helsinki, Finland; Department of Forest Sciences/Institute for Atmospheric and Earth System Research (INAR), University of Helsinki, P.O. Box 68, FI-00014 Helsinki, Finland; Ecosystems and Modelling, Bioeconomy and Environment, Natural Resources Institute Finland (Luke), Latokartanonkaari 9, FI-00790 Helsinki, Finland; Stable Isotope Laboratory of Luke (SILL), Natural Resources Institute Finland (Luke), Latokartanonkaari 9, FI-00790 Helsinki, Finland; Faculty of Biological and Environmental Sciences, Viikki Plant Science Centre, University of Helsinki, P.O. Box 68, FI-00014 Helsinki, Finland; Faculty of Biological and Environmental Sciences, Viikki Plant Science Centre, University of Helsinki, P.O. Box 68, FI-00014 Helsinki, Finland; Department of Environmental Sciences—Botany, University of Basel, Schönbeinstrasse 6, 4056 Basel, Switzerland; Ecosystems and Modelling, Bioeconomy and Environment, Natural Resources Institute Finland (Luke), Latokartanonkaari 9, FI-00790 Helsinki, Finland; Ecosystems and Modelling, Bioeconomy and Environment, Natural Resources Institute Finland (Luke), Latokartanonkaari 9, FI-00790 Helsinki, Finland; Stable Isotope Laboratory of Luke (SILL), Natural Resources Institute Finland (Luke), Latokartanonkaari 9, FI-00790 Helsinki, Finland; Department of Forest Sciences/Institute for Atmospheric and Earth System Research (INAR), University of Helsinki, P.O. Box 68, FI-00014 Helsinki, Finland; Department of Environmental Sciences—Botany, University of Basel, Schönbeinstrasse 6, 4056 Basel, Switzerland; Center of Microbiology and Environmental Systems Science, Division of Terrestrial Ecosystem Research, University of Vienna, Djerassiplatz 1, 1030 Vienna, Austria; Ecosystems and Modelling, Bioeconomy and Environment, Natural Resources Institute Finland (Luke), Latokartanonkaari 9, FI-00790 Helsinki, Finland; Stable Isotope Laboratory of Luke (SILL), Natural Resources Institute Finland (Luke), Latokartanonkaari 9, FI-00790 Helsinki, Finland; Faculty of Science, Institute for Atmospheric and Earth System Research (INAR)/Physics, University of Helsinki, P.O. Box 68, FI-00014 Helsinki, Finland; College of Urban and Environmental Sciences, Peking University, Haidian District, Beijing 100871, China; Ecosystems and Modelling, Bioeconomy and Environment, Natural Resources Institute Finland (Luke), Latokartanonkaari 9, FI-00790 Helsinki, Finland; Stable Isotope Laboratory of Luke (SILL), Natural Resources Institute Finland (Luke), Latokartanonkaari 9, FI-00790 Helsinki, Finland

**Keywords:** boreal forest, carbon isotope, intra-seasonal, oxygen isotope, relative humidity, Scots pine (*Pinus sylvestris*)

## Abstract

Interpreting tree-ring oxygen isotope composition (δ^18^O_ring_) is complicated by the combined influences of source water (δ^18^O_sw_) and relative humidity (RH). This study investigates intra- and interannual δ^18^O_ring_ signals in Scots pine stands in southern and northern Finland over a 10-year period (2010–19). We applied correlation analysis and process-based intra-annual δ^18^O and δ^13^C modeling to disentangle RH and δ^18^O_sw_ signals in δ^18^O_ring_. Growth models for xylogenesis were used to date the analyzed tree-ring subsections. Dual isotope modeling provided additional constraints to evaluate the uncertainties caused by xylogenesis. Our results show that δ^18^O_ring_ signals were dominated by RH, due to its much higher relative variability compared with that of δ^18^O_sw_. Generally, correlations were stronger at interannual than intra-annual resolution. Modeling indicated that additional factors complicate the interpretation of intra-annual δ^18^O_ring_ signals beyond the combined effects of RH and δ^18^O_sw_. We show that seasonal variations in the proportion of oxygen exchange with source water during the pathway to tree-ring cellulose may explain the lower RH signal at intra-annual resolution. Incorporating variable oxygen exchange improved model performance and aligned modeled δ^18^O_ring_ more closely with observations. Despite the encouraging modeling results using growth models for dating tree-ring subsections, we recognize that time integration and alignment will continue to challenge the interpretation of intra-annual isotope signals. Our study demonstrates that combining empirical data analysis with mechanistic modeling is essential for resolving the environmental drivers of δ^18^O_ring_ and for extending interpretations beyond site-specific conditions. Our findings are particularly relevant as intra-annual δ^18^O analysis becomes more common, underscoring the importance of time integration and dating tree-ring subsections, highlighting future research needs (e.g., varying oxygen exchange) and advancing their use for climate reconstruction.

## Introduction

The oxygen isotope compositions of tree rings (δ^18^O_ring_) are a valuable archive for assessing past environmental conditions and tree physiological responses (McCarroll and Loader 2004). δ^18^O_ring_ values offer insights into long-term precipitation patterns (Rinne et al. 2013, Loader et al. 2020, Treydte et al. 2024, 2006), relative humidity (RH) or vapor pressure deficit (Kahmen et al. 2011, Wernicke et al. 2015, Xu et al. 2022), plant water sources (Berkelhammer et al. 2020, Churakova-Sidorova et al. 2020, Bailey et al. 2023) and soil moisture (Wang et al. 2021). While conventionally investigated at annual resolution, the exploration at intra-annual timescales has recently become more common (Belmecheri et al. 2022, Leavitt and Szejner 2022, Martínez-Sancho et al. 2023). Intra-annual δ^18^O_ring_ records, obtained by measuring δ^18^O of different segments within a single tree ring, offer insights into environmental conditions at intra-annual timescales (Berkelhammer and Stott 2009, Roden et al. 2009, Szymczak et al. 2019). This increased focus on intra-annual resolution highlights the need to address the challenges of predicting plant responses across varying temporal and spatial scales, where processes observed at one resolution may not directly translate to another (Evans et al. 2025).

The two main factors defining temporal variability of δ^18^O_ring_ are: (i) the evaporative ^18^O-enrichment of water (‘evaporative enrichment’ hereafter for brevity) occurring at the leaf level and (ii) source water δ^18^O (δ^18^O_sw_), which depends on a combination of hydroclimatic conditions determining the isotopic composition of precipitation, local soil hydrological processes and root uptake distribution (Roden et al. 2000, Ogée et al. 2009, Yoshimura et al. 2011). At the leaf level, δ^18^O_sw_ is exposed to evaporative enrichment, which is primarily associated with variations in atmospheric water demand, as described by the Craig–Gordon model (Craig and Gordon 1965). This model, describing isotopic enrichment at the evaporative sites within the leaf, has been adapted to account for the mixing of enriched evaporative water and unenriched xylem water (Leaney et al. 1985, Farquhar and Lloyd 1993, Barbour et al. 2000, Baca Cabrera et al. 2023). Leaf sugars, carrying the evaporative enrichment signal through the phloem, serve as the substrate for cellulose synthesis in the cytoplasm of xylem cells (Barbour et al. 2000, Cernusak et al. 2005). During the pathway to the tree ring, a proportion of oxygen atoms from the sugars exchanges with surrounding water at the site of cellulose synthesis (Sternberg et al. 1986) and plausibly also during phloem loading and transport (Gessler et al. 2013, Fiorella et al. 2022, Pan et al. 2024). In the tree stem, this water is generally assumed to be composed of unenriched xylem water (δ^18^O_sw_) only, i.e., the so-called *p*_x_ factor is assumed to be 1 (Cernusak et al. 2005, Sternberg and Ellsworth 2011). As a result of oxygen exchange, the δ^18^O_ring_ is a mixture of leaf sugars δ^18^O and source water δ^18^O_sw_ signals.

Despite a well-established theoretical framework, significant gaps in the understanding of how the isotopic composition of photosynthates and δ^18^O_sw_ are integrated into the wood remain (Gessler et al. 2014). We identified three major challenges, which especially hinder the interpretation of environmental signals underlying the intra-annual δ^18^O_ring_ records. First, both leaf evaporative enrichment, driven by RH, and δ^18^O_sw_ may show seasonal trends that correlate with each other within the growing season. When mixed on the pathway to tree-ring cellulose, opposing trends in leaf sugar δ^18^O and δ^18^O_sw_ can result in the cancellation of RH or δ^18^O_sw_ signals in intra-annual δ^18^O_ring_ (Szejner et al. 2025). Second, interpreting δ^18^O signals of intra-annual tree-ring subsections requires knowledge of their formation periods to match them with representative environmental data (Gessler et al. 2009, Ogée et al. 2009, Pérez-de-Lis et al. 2022). The growth periods of tree-ring subsections can be derived from direct measurements of wood growth dynamics using repeated microcore sampling (Rinne et al. 2015, Martínez-Sancho et al. 2023). However, when interpreting intra-annual δ^18^O_ring_ signals over periods longer than a few years, particularly in the absence of extensive measurement campaigns, we need to rely on simple monthly averaging (Szymczak et al. 2019, Watanabe et al. 2023) or preferably on growth models that have been calibrated using xylogenesis data (Schiestl-Aalto et al. 2015, Friend et al. 2022, Tang et al. 2023). Lastly, the assumption of a constant fraction of oxygen exchange with surrounding water is increasingly debated (Song et al. 2014, Belmecheri et al. 2018, Szejner et al. 2020, Martínez-Sancho et al. 2023). Factors driving the seasonal variation of oxygen exchange remain an active area of research, warranting further investigation (Song et al. 2022). Process-based isotope-enabled models predicting intra-annual δ^18^O_ring_ (Ogée et al. 2009, Zeng et al. 2017) present a still underused tool to investigate these identified challenges, especially when constraining models with both δ^18^O and δ^13^C.

This study focuses on these challenges and their effects on the interpretation of environmental signals of δ^18^O_ring_ across temporal scales. The specific objectives are:

(i) To characterize variation of intra-annual δ^18^O_ring_.
(ii) To evaluate the strength δ^18^O_sw_ and RH signals in δ^18^O_ring_ across inter- and intra-annual timescales using a growth model to date wood samples.
(iii) To address the uncertainties regarding oxygen exchange with surrounding water and the integration of δ^18^O signals over intra-annual timescales into tree rings.

To address these aims, we measure 10-year-long intra-annual δ^18^O_ring_ records from two boreal Scots pine stands in southern and northern Finland. We combine statistical analysis with dynamic process–based isotope modeling, which mechanistically describes the formation of the δ^18^O and δ^13^C signals from leaves and to tree rings (Ogée et al. 2009, Leppä et al. 2022). A separate model is used to predict δ^18^O_sw_ (Leppä et al. 2022) based on precipitation δ^18^O (Yoshimura et al. 2011, 2008). Both statistical and modeling approaches rely on growth models for xylogenesis (Schiestl-Aalto et al. 2015) to date the analyzed tree-ring subsections. The isotope model is applied to complement the statistical analysis and to address the third objective. Including δ^13^C modeling alongside δ^18^O provides additional constraints to the model, especially regarding uncertainties related to time integration. Intra-annual δ^13^C_ring_ data from the same sites and years were available from an earlier study (Tang et al. 2023).

## Materials and methods

### Study sites and environmental data

The study focused on two Scots pine (*Pinus sylvestris* L.) dominated forests: Hyytiälä in southern Finland (61°51′N, 24°17′E) and Värriö in northern Finland (67°46′N, 29°35′E). Both sites belong to the Integrated Carbon Observation System (ICOS) and FluxNet networks. Hyytiälä is a managed, mixed coniferous forest with ca 60-year-old stand of ca 20 m-tall trees (1340 trees ha^−1^). Värriö, located close to the northern altitudinal tree line, is a sparser, unmanaged forest with a 60-year-old Scots pine stand of ca 10 m in height (750 trees ha^−1^). The long-term (1991–2020) mean annual temperature is 4.1 °C at Hyytiälä and 0.1 °C at Värriö, with mean annual precipitation of 690 and 607 mm and RH 78% and 79%, respectively (Jokinen et al. 2021). Both sites are located on well-drained mineral soils.

Half-hourly meteorological and environmental data for the study period 2010–19 used in the data analysis (see section Data analysis), and for running the growth model (Schiestl-Aalto et al. 2015) and isotopic model (see section Modeling intra-annual tree-ring δ^18^O) were available for both sites on the AVAA Smart SMEAR portal (https://smear.avaa.csc.fi/) and https://etsin.fairdata.fi/ (Aalto et al. 2023, 2024). Additionally, we used δ^18^O of water vapor and δ^18^O of precipitation predicted by the isotope-enabled, nudged atmospheric general circulation model IsoGSM available at a horizontal resolution of ~200 km and with a timestep of 6 h (Yoshimura et al. 2011, 2008), and δ^13^C of atmospheric CO_2_ available at weekly resolution from Pallas-Sammaltunturi GAW-station (Michel et al. 2025).

### Sampling and isotope analysis

Tree core samples were collected at ca 1.3 m height after the growing season in 2019 from five mature trees per site. The tree cores were resin extracted in a Soxhlet using a 2:1 ethanol and toluene solution to remove resins, oils and other compounds, which are mobile and contain a muted, compositionally distinct δ^18^O signal compared with the structural polymers cellulose and lignin (Tang et al. 2024). Tree rings from 2010 to 2019 were then dissected into thin sections (ca 160 μm) using a cryo-microtome. This thickness was chosen to provide sufficient material for accurate δ^18^O analysis (Belmecheri et al. 2022). In cases where tree rings were at their narrowest, only as few as 2 sections could be collected per ring, as opposed to widest rings where as many as 15 subsections were analyzed. On average, 10 and 8 thin sections per tree ring were collected at Hyytiälä and Värriö, respectively.

The resin-extracted wood samples from Hyytiälä were analyzed for δ^18^O at the stable isotope ecology laboratory at the University of Basel, while Värriö samples were analyzed in the University of Vienna. In both laboratories, dry samples were packed into silver capsules and introduced to a Flash isotope ratio mass spectrometry (IRMS) analyzer using an autosampler. Samples were flushed with helium and allowed to equilibrate with the analyzer prior to measurement. The sample reactor was operated using glassy carbon pyrolysis and was interfaced with a Delta V Plus IRMS via a ConFlo IV, controlled by Isodat 3.0 software. Sample nitrogen was prevented from entering the ion source using gas dilution in the ConFlo IV, avoiding δ^18^O measurement bias due to nitric oxide formation (Brand et al. 2009). Measured δ^18^O values were normalized to the Vienna Standard Mean Ocean Water/Standard Light Antarctic Precipitation (VSMOW/SLAP) reference δ-scale using in-house dimethyl-, trimethyl- and benzoic acid standards. Long-term analytical precision for both labs was ≤0.2‰, based on repeat measurements of a benzoic acid quality control sample.

For the years 2018–19, δ^18^O data were also obtained from rainwater, twig water (i.e., source water), leaf water, leaf water-soluble carbohydrates (WSCs, i.e., sugars and sugar alcohols, here mainly pinitol) and stem phloem WSC. These data were used to evaluate the inputs and outputs of the isotope model (see section Modeling intra-annual tree-ring δ^18^O), which was already done at the leaf level for Hyytiälä by Leppä et al. (2022). In short, cumulative rainwater was collected monthly, current and 1-year-old needles and their corresponding twigs were collected from the sunlit canopy of five mature trees per site weekly for WSC sampling and six to nine times per growing season for water sampling, and stem phloem samples were collected with a 2-cm diameter metal puncher at 1.3 m height from the same trees six times per growing season and site. Further details on sampling and isotope analysis are given in Leppä et al. (2022) and Szejner et al. (2025).

For δ^13^C_ring_, the intra-annual variation in tree-ring δ^13^C (resin-extracted wood) was determined by laser ablation IRMS analysis (Tang et al. 2023). The ablated cores were the same, which were later dissected for δ^18^O analysis. In short, as many as possible 40 μm-wide analysis lines were fitted on each tree ring to obtain high-resolution δ^13^C_ring_ data. The results were normalized against USGS-55 (δ13C = −27.13‰) and yucca plant (in-house reference material, δ^13^C = −15.46‰). IAEA-C3 cellulose was analyzed as a quality control, returning the δ^13^C value of −24.64 ± 0.25‰ (*n* = 347) (expected δ^13^C = −24.91 ± 0.49‰). The intra-annual resolution δ^13^C_ring_ was higher than for δ^18^O_ring_. On average, 15 and 9 laser tracks per tree ring were ablated for Hyytiälä and Värriö, respectively.

### Averaging and dating tree-ring subsections

**Figure 1:**
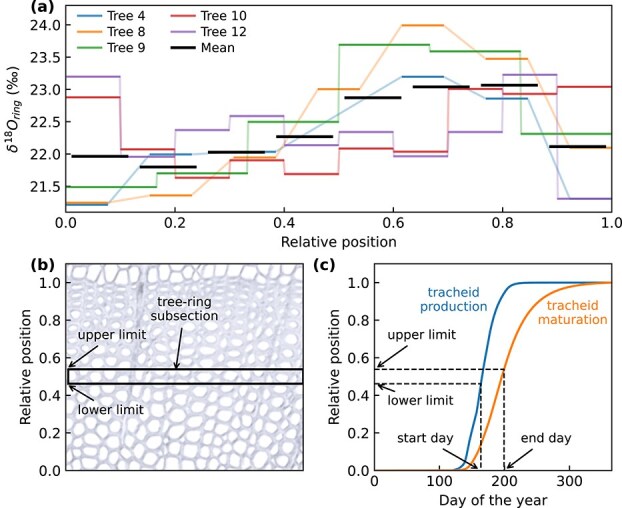
Example of (a) calculating mean tree-ring δ^18^O (δ^18^O_ring_) series using data from five trees, (b) relative position of sampled tree-ring subsection and (c) applying the xylogenesis growth curves for tracheid production and maturation to obtain the time window corresponding to the tree-ring subsection. In (a), horizontal bars show the relative fraction within the ring that each tree-ring subsection represent and semitransparent lines show the interpolated series. The resolution of the mean tree-ring δ^18^O_ring_ series was selected based on the mean number and relative length of subsections of individual trees for each year.

Following the sampling, the dataset consisted of five δ^18^O_ring_ and δ^13^C_ring_ series from individual trees for both sites with varying amounts of measurements, as the number and coverage of tree-ring subsections varied between isotopes, individual trees and rings. To obtain δ^18^O_ring_ and δ^13^C_ring_ series representing the average of the trees for each site, we applied the following averaging approach (example shown in Figure 1a). First, each tree’s isotope series was defined at 0.001 resolution (relative position within ring): values within subsections were assigned the measured isotope value, values between subsections were linearly interpolated and values at beginning and end were filled with first and last subsection’s isotope value, respectively (Figure 1a). Second, for each isotope, studied year and site, we selected the resolution of the mean series based on the mean number and relative length of subsections. Finally, mean δ^18^O_ring_ and δ^13^C_ring_ series for both sites were calculated as the average of the five trees’ values residing within the mean series subsections (Figure 1a). By selecting the resolution of the averaged series for each year and site separately instead of for the whole dataset, we expect to minimize biases arising from aggregating measurements representing different length fractions of the rings.

To define the time window corresponding to each of the analyzed tree-ring subsections, we estimated xylogenesis for each site using the dynamic growth model, Carbon Allocation Sink Source Interaction (CASSIA) (Schiestl-Aalto et al. 2015, Tang et al. 2023). The modeled growth curves, defining the timing for tracheid formation and maturation as relative position within the tree ring (Figure 1c), were compared against xylogenesis curves derived from micro-core data for years 2007, 2008, 2009, 2018 and 2019, for both sites (Figure S1 available as Supplementary Data at *Tree Physiology* Online). Uncertainties in growth timing were addressed by analyzing correlations and model fit with formation and maturation dates shifted by ±20 days. The growth curves enabled us to identify the start and end days for each tree-ring subsection, defining a specific time window for each wood sample (Figure 1b and c). End dates were limited to the dates corresponding to the relative position 0.97 within the tree ring, as modeled dates for full ring maturation (relative position 1.0) extended far into the winter (Figure 1c). The length of the time windows for the mean series was, on average, 44 days (min–max: 21–101) and 34 days (23–60) for δ^18^O_ring_ and 40 days (min–max: 17–96) and 29 days (21–50) for δ^13^C_ring_ in Hyytiälä and Värriö, respectively.

### Data analysis

We performed correlation analysis on the data to investigate the main drivers of δ^18^O_ring_, i.e., δ^18^O_sw_ (modeled based on rootzone isotope budget; see Methods S1) and RH. We calculated δ^18^O_sw_ and RH corresponding to each tree-ring subsection by using the time window determined from xylogenesis (see section Averaging and dating tree-ring subsections). The RH and δ^18^O_sw_ values were calculated from half-hourly data weighted by modeled net CO_2_ exchange (*A*_n_) to account for weighting within a day (relevant for RH only) and weighting between days along the assigned time window. For comparison, we calculated RH and δ^18^O_sw_ values using a fixed daytime window (9 a.m. to 3 p.m.) and weighting based on clear-sky global radiation (function of day of year and zenith angle). The latter two do not rely on the availability of meteorological data or leaf gas exchange modeling, making them more widely applicable.

Pearson correlations between δ^18^O_ring_ and the two environmental drivers (δ^18^O_sw_ and RH) were calculated for individual trees, for the δ^18^O_ring_ series representing the mean of trees per site and across different temporal scales: including (i) whole timeseries (10-year intra-annual), (ii) data averaged by year (interannual) and (iii) yearly averaged data grouped by early growing season (interannual EGS), mid-growing season (interannual MGS) and late growing season (interannual LGS) based on their time window center-point date. The periods corresponding to EGS, MGS and LGS were defined as mid-May to mid-June, mid-June to mid-August and mid-August to September, respectively, for Hyytiälä; and June, July and August, respectively, for Värriö.

### Modeling intra-annual tree-ring **δ**^18^O

**Figure 2:**
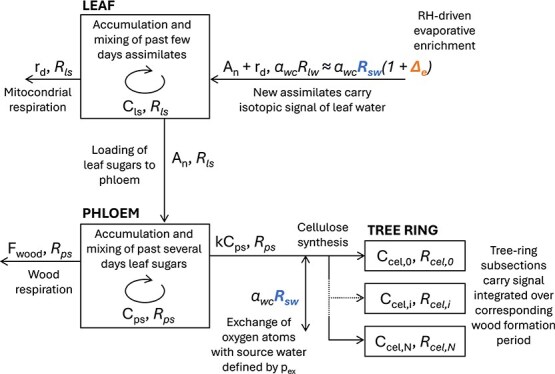
Conceptual figure of oxygen isotope fractionation model tracing δ^18^O of sugars in the leaf and phloem pools, and finally into cellulose of tree-ring subsections (*i* = 0 …*N*), showing the sources of relative humidity (evaporative enrichment, Δ_e_) and source water (*R*_sw_) signals. Each arrow and pool are denoted by their sugar flux or concentration, respectively, followed by the isotopic ratio of that flux or pool (in italic). *A*_n_, *r*_d_, *F*_wood_ and *kC*_ps_ are net CO_2_ exchange, mitochondrial respiration, wood respiration and the use of sugars as substrate for biomass synthesis, respectively; *C*_ls_, *C*_ps_ and *C*_cel,i_ are the concentrations of carbon in the leaf sugar pool, the phloem sugar pool and the cellulose of tree-ring subsection *i*, respectively; *R*_sw_, *R*_lw_, *R*_ls_, *R*_ps_ and *R*_cel,i_ are the oxygen isotope ratios of source water, leaf water, leaf sugar, phloem sugar and cellulose of tree-ring subsection *i*, respectively; and *α*_wc_ (=1 + *ε*_wc_) is the biochemical fractionation factor.

We applied the leaf-level isotopic fractionation model of Leppä et al. (2022) to predict needle sugar δ^18^O, which is here further traced down to the phloem sugar pool and finally to cellulose of tree-ring subsections following Ogée et al. (2009). In short, the model is comprised of two well-mixed pools, the leaf pool and the phloem pool, and an archive of tree-ring subsections (Figure 2). The leaf pool is fed by new assimilates that carry the isotopic signal of leaf water, a combination of signals from source water and evaporative enrichment. The leaf sugar pool is used as the substrate for mitochondrial respiration and loaded into the phloem. From the phloem, pool sugars are used for biomass synthesis and as a substrate for wood respiration. During cellulose synthesis, oxygen atoms are exchanged with source water. A proportion of the new biomass is allocated to tree-ring subsections, depending on the time window defined by xylogenesis for each subsection. As in Ogée et al. (2009), the model neglects starch storage and re-mobilization. The use of previous-year reserves has been reported as negligible based on intra-annual δ^13^C_ring_ data for our study sites (Tang et al. 2023) as well as for other conifers, including Norway spruce (Marshall and Linder 2013) and larch (Rinne et al. 2015). The following model equations are expressed in terms of isotopic ratios (*R* = ^18^O/^16^O), which can be converted to ‘delta’ notation:


(1) \begin{equation*} {\delta}^{18}O=\left(\frac{R}{R_{std}}-1\right) \end{equation*}

where *R*_std_ is the ^18^O/^16^O isotope ratio defined by VSMOW.

The model by Leppä et al. (2022) builds on a photosynthesis model, which provides the necessary inputs for modeling leaf sugar δ^18^O. The changing isotope ratio of leaf water (*R*_lw_) over time is computed following the nonsteady-state formulation (Farquhar and Cernusak 2005):


(2) \begin{equation*} \frac{d\left(W{R}_{lw}\right)}{dt}=\frac{E{w}_i}{\alpha^{+}{\alpha}_k\left({w}_i-{w}_a\right)}\left({R}_{lw, ss}-{R}_{lw}\right) \end{equation*}

where *W* (mol m^−2^) is leaf water content, *E* (mol m^−2^ s^−1^) is the transpiration rate and *α*^+^ (= 1 + *ε*^+^) is temperature-dependent equilibrium fractionation during vaporization (Majoube 1971); *w*_a_ and *w*_i_ (mol mol^−1^) are the mole fractions of water vapor in the atmosphere and inside the leaf, respectively. *α*_k_ (−) describes the kinetic isotope fractionation during water vapor diffusion through stomata and the leaf boundary layer (Farquhar et al. 1989):


(3) \begin{equation*} {\alpha}_k=1+\frac{g_b{\varepsilon}_{ks}+{g}_s{\varepsilon}_{kb}}{g_b+{g}_s} \end{equation*}

where *g*_s_ and *g*_b_ (mol m^−2^ s^−1^) are stomatal and boundary layer conductance, respectively, and *ε*_ks_ and *ε*_kb_ are fractionation factors associated with water vapor transport through stomata and boundary layer, respectively. The oxygen isotope ratio of leaf water under steady state (*R*_lw,ss_) is computed as (Flanagan et al. 1991):


(4) \begin{equation*} {R}_{lw, ss}={\alpha}^{+}\left({\alpha}_k\;\frac{w_i-{w}_a}{w_i\;}{R}_{sw}+\frac{w_a}{w_i}{R}_v\right) \end{equation*}

where *R*_v_ and *R*_sw_ are the isotopic ratios of oxygen in atmospheric water vapor and source water, respectively. These were not directly measured at the study sites for the whole study period. Therefore, we used δ^18^O of water vapor and δ^18^O of precipitation derived from IsoGSM outputs (Yoshimura et al. 2011, 2008). Comparison between monthly measured and IsoGSM precipitation δ^18^O (2018–19) showed a consistent offset, which led to the decision to apply site-specific corrections to IsoGSM outputs, 2.99‰ and 3.34‰ for Hyytiälä and Värriö, respectively (Figure S2a available as Supplementary Data at *Tree Physiology* Online). The resulting precipitation δ^18^O was further used as input for a rootzone isotope budget model to obtain δ^18^O_sw_ (Methods S1; Figure S2b available as Supplementary Data at *Tree Physiology* Online). Equations (2) and (4) are based on the traditional Craig–Gordon model, which commonly overestimates leaf water evaporative enrichment. Correction factors based on the Péclet effect (Farquhar and Lloyd 1993) or the two-pool model (Leaney et al. 1985) were, however, neglected here as Leppä et al. (2022) showed that they had only a marginal role on leaf water δ^18^O in Hyytiälä.

The isotopic ratio of the leaf sugar pool is solved from (Figure 2; Leppä et al. 2022):


(5) \begin{equation*} \frac{d\left({C}_{ls}{R}_{ls}\right)}{dt}=\left({A}_n+{r}_d\right){\alpha}_{wc}{R}_{lw}-{r}_d{R}_{ls}-{A}_n{R}_{ls} \end{equation*}

where *R*_ls_ is the isotopic ratio in the leaf sugar pool, *C*_ls_ (μmol of C m^−2^) sugar concentration in the leaf, *α*_wc_*R*_lw_ is the isotopic ratio of new assimilates (Barbour et al. 2000) and *A*_n_ and *r*_d_ (μmol m^−2^ s^−1^) are the modeled net CO_2_ exchange and mitochondrial respiration, respectively. The last term in Eq. (5) defines the discharge from the leaf into the phloem. Leppä et al. (2022) showed *C*_ls_ to be relatively constant in time; thus, the rate of discharge is set equal to *A*_n_. The biochemical fractionation factor (*α*_wc_ = 1 + *ε*_wc_) represents the fractionation associated with oxygen isotope exchange between carbonyl oxygen and the water from which it was synthesized (Sternberg et al. 1986). We defined *ε*_wc_ as temperature-dependent following Sternberg and Ellsworth (2011), who reported a particularly strong incline in *ε*_wc_ for temperatures below 20 °C. Leppä et al. (2022) showed that in the cool boreal conditions of Hyytiälä, the temperature-dependent *ε*_wc_ improved model performance notably compared with the commonly used constant value 27‰.

From leaves, sugars flow downstream to a well-mixed phloem sugar pool (Figure 2). The carbon budget of this pool was adopted from Ogée et al. (2009), who described the tree’s nonstructural carbon pool as:


(6) \begin{equation*} \frac{d{C}_{ps}}{dt}={A}_n-{F}_{wood}-k{C}_{ps} \end{equation*}

where *C*_ps_ (μmol of C m^−2^) is the concentration of phloem sugar, *A*_n_ (μmol of C m^−2^ s^−1^) defines the inflow from leaves, *F*_wood_ (μmol of C m^−2^ s^−1^) is woody respiration, and the term *kC*_ps_ defines the use of sugars as a substrate for biomass synthesis, where *k* (s^−1^) is a constant rate coefficient. *F*_wood_ was set to 0.25rd based on the measured component CO_2_ fluxes in Hyytiälä (Kolari et al. 2009). Note that m^2^ in the units refers to leaf area (or ‘active leaf area’ as not all leaves photosynthesize at the same rate) and hereby, we do not need to upscale leaf-level inputs to tree-level. The associated oxygen isotope phloem budget is (Ogée et al. 2009):


(7) \begin{equation*} \frac{d\left({C}_{ps}{R}_{ps}\right)\;}{dt}={A}_n{R}_{ls}-{F}_{wood}\left(1-e\right){R}_{ps}-k{C}_{ps}\left(1-x\right){R}_{ps} \end{equation*}

where *R*_ps_ is the isotopic ratio of oxygen in the phloem sugar pool and *e* and *x* are isotope fractionations associated with wood respiration (relevant only for δ^13^C) and biomass synthesis, respectively. The oxygen isotope ratio in cellulose of the tree-ring subsection *i* (*R*_cel,i_; Eq. (8); Figure 2) is a mix of the isotopic ratio in source water (first term) and that of phloem sugars (second term) integrated over the tracheid formation to maturation period (Ogée et al. 2009):


(8) \begin{equation*} {R}_{cel,i}={p}_{ex}\frac{\int{\alpha}_{wc}{R}_{sw}{C}_{ps} dt\ }{\int{C}_{ps} dt} + \left(1-{p}_{ex}\right)\frac{\int{R}_{ps}{C}_{ps} dt}{\int{C}_{ps} dt} \end{equation*}

where the integrals are computed over tracheid formation and maturation periods for the tree-ring subsection *i* (available from the xylogenesis data, see section Averaging and dating tree-ring  subsections). The parameter *p*_ex_ characterizes the proportion of oxygen atoms exchanged with source water during cellulose synthesis (Sternberg et al. 1986).

Alongside δ^18^O, we modeled intra-annual δ^13^C_ring_. A description of the leaf-level δ^13^C model can be found in Leppä et al. (2022). From leaves to tree-ring cellulose, Eqs (6) and (7) apply also to δ^13^C and Eq. (8) simplifies to (Ogée et al. 2009):


(9) \begin{equation*} {R}_{cel,i}=\frac{\int{R}_{ps}{C}_{ps} dt}{\int{C}_{ps} dt} \end{equation*}

The model was applied to both sites using half-hourly meteorological/environmental data (air temperature, atmospheric CO_2_ and water vapor, photosynthetically active radiation and volumetric soil moisture) and isotopic data (*R*_v_, *R*_sw_, δ^13^C of atmospheric CO_2_) for 2010–19. The model was used to predict δ^18^O_ring_ and δ^13^C_ring_ series corresponding to the observed average tree-ring isotope series (see section Averaging and dating tree-ring subsections). Parameters applied in the isotopic simulations are listed in Table 1 (for parameters of the photosynthesis and leaf-level δ^13^C model, see Leppä et al. (2022)). Parameters *k* and *p*_ex_ were selected by calibration. We ran the model for both sites with *k* ranging from 1 to 50 year^−1^. For Hyytiälä, correlations between modeled and measured δ^18^O_ring_ and δ^13^C_ring_ both peaked around *k* = 30. For Värriö, an optimum *k* was not identifiable based on the same approach, but correlations between measured and modeled phloem WSC suggested *k* = 30 was also suitable for Värriö. *p*_ex_ was selected to minimize the mean absolute error (MAE) between modeled and measured δ^18^O_ring_, which at both sites resulted in *p*_ex_ = 0.36. Hereby, identical parameters were used for both sites.

**Table 1:** Parameters for isotopic modeling

Parameter^1^	Description	Value	Source
W (mol m^−2^)	Leaf mesophyll water volume	5.6	Hyytiälä measurements, see Leppä et al. (2022)
*ε* ^+^(−)	Equilibrium fractionation during vaporization	temperature dependent	Majoube (1971)
*ε*_kb_ (−)	Fractionation during diffusion of water vapor through boundary layer	19‰	Merlivat (1978)
*ε*_ks_ (−)	Fractionation during diffusion of water vapor through stomata	28‰	Merlivat (1978)
*ε*_wc_ (−)	Biochemical fractionation factor	temperature dependent	Figure 1 in Sternberg and Ellsworth (2011)
*C*_ls_ (μmol of C m^−2^)	Concentration of leaf sugar	1.96 × 10^5^	Hyytiälä measurements, see Leppä et al. (2022)
*k* (year^−1^)	Turnover rate of phloem sugar pool	30	Calibrated
*x* (−)^2^	Fractionation during wood formation	0‰	Ogée et al. (2009)
*e* (−)^3^	Fractionation during wood respiration	0‰	Ogée et al. (2009)
*p*_ex_ (−)	Proportion of exchangeable oxygen during cellulose synthesis	0.36	Calibrated

^1^Parameter units are given in parenthesis, where ‘–’ stands for unitless and all area-based units refer to all-sided leaf area.

^2^Can be defined separately for δ^18^O and δ^13^C.

^3^Only relevant for δ^13^C.

As the model predicts isotope values of cellulose, we applied offsets −4.4‰ and −1.1‰ for δ^18^O and δ^13^C, respectively, to obtain isotope values of resin-extracted wood based on unpublished annual data from Hyytiälä 1969–2018 (standard deviations of mean offsets 0.7‰ and 0.3‰ for δ^18^O and δ^13^C, respectively). While cellulose is generally recognized to have a more direct and interpretable isotope signal (Helle et al. 2022), studies have shown strong correlations between cellulose and resin-extracted wood δ^18^O and δ^13^C (Barbour et al. 2001, Szymczak et al. 2011, Weigt et al. 2015, Guerrieri et al. 2017), supporting the use of resin-extracted wood. To quantify the uncertainty introduced by potential variation in cellulose-to-lignin ratios, we carried out simple two-pool mass-balance calculations (Schleser et al. 2015; Methods S2). Assuming typical conifer sapwood composition with 75% of cellulose and 25% of lignin and allowing the lignin fraction to vary by ±5 percentage points (Walcroft et al. 1997), we estimated the uncertainty caused by varying lignin fraction to be ±1.0‰ and ± 0.2‰ for δ^18^O and δ^13^C, respectively. The calculations are based on constant isotope offsets between cellulose and lignin, but as there are some indications that this may not always hold (Helle et al. 2022), this limits our uncertainty estimates.

Model comparison against leaf and stem phloem sampling data collected during 2018–19, suggested that the model captures well the observed variations of δ^18^O in different pools before being incorporated into tree rings (Figure S2 available as Supplementary Data at *Tree Physiology* Online). The correlation between modeled and measured needle water δ^18^O was *r* = 0.96–0.98 (MAE = 1.5–2.8‰), needle WSC δ^18^O *r* = 0.90 (MAE = 1.0–1.2‰), and phloem WSC δ^18^O *r* = 0.75–0.93 (MAE = 0.7–1.0‰), which gives a good basis for applying the model further for analyzing δ^18^O_ring_. Note that observations were for needle and phloem WSC, not purely sugars, for which the above model equations are given. As in Leppä et al. (2022), leaf WSC was assumed to differ in δ^18^O compared with leaf sugars due to a constant presence of more ^18^O-depleted pinitol (measured *C*_pinitol_ = 0.7*C*_ls_, assumed δ^18^O_pinitol_ = 25‰). The same assumption seemed valid for Värriö as well (Figure S2d available as Supplementary Data at *Tree Physiology* Online). The comparison between modeled and measured phloem WSC was done here for the first time for both sites (Figure S2e available as Supplementary Data at *Tree Physiology* Online). We found the need to account for (i) a constant pinitol presence in time (concentration ~0.15*C*_ps_ during mid-growing season according to measurements at Hyytiälä, and assumed δ^18^O_pinitol_ = 25‰) and (ii) the exchange of oxygen atoms of sugars with source water during phloem loading at a rate of 3/11 as suggested for Scots pine by Gessler et al. (2013). While there remain great uncertainties on possible isotopic fractionation during phloem loading, the latter provided a significant correction to the level of modeled phloem WSC (Figure S2e available as Supplementary Data at *Tree Physiology* Online). For δ^18^O_ring_, it is irrelevant whether the exchange with source water happens during phloem loading in addition to cellulose synthesis, if *p*_ex_ > 3/11 and we assume the same oxygen atoms at more readily exchanged positions (Waterhouse et al. 2013) are involved in both processes (unchanged fraction, 1 — *p*_ex_, remains the same). So, to keep the model equations simple, exchange during phloem loading was only accounted for in the comparison to phloem WSC data, not Eq. (7) or modeled δ^18^O of phloem sugars presented later in this manuscript.

## Results

### Relative humidity and source water **δ**^18^O signals in observed tree-ring **δ**^18^O

**Figure 3:**
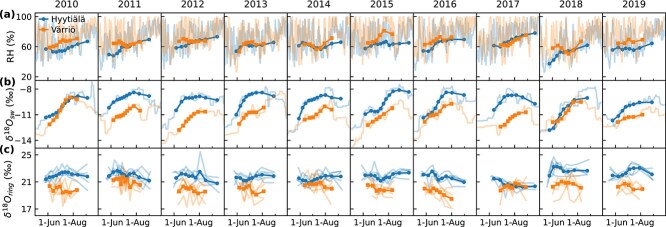
Variability of (a) relative humidity (RH), (b) δ^18^O of source water (δ^18^O_sw_) and (c) mean tree-ring δ^18^O (δ^18^O_ring_) of trees for Hyytiälä and Värriö over the growing seasons 2010–19. Lines with markers for RH and δ^18^O_sw_ are integrated over time windows corresponding to growth periods of tree-ring subsections and weighted by modeled net CO_2_ exchange (A_n_). The semitransparent lines represent (a) daily daytime RH, (b) daily δ^18^O_sw_ and (c) measured δ^18^O_ring_ of tree-ring subsections of individual trees.

Timeseries of RH, δ^18^O_sw_ and observed mean δ^18^O_ring_ from Hyytiälä and Värriö revealed consistent seasonal patterns during 2010–19. Relative humidity had an increasing seasonal trend, with high day-to-day variability, at both sites (Figure 3a). δ^18^O_sw_ typically increased in the beginning of the growing season, followed by a decrease in the fall (Figure 3b, semitransparent lines), which reflected the seasonal pattern of precipitation δ^18^O with a few weeks’ lag, depending on the amount of precipitation and soil moisture conditions. However, in Värriö, the autumn decline in δ^18^O_sw_ occurred only after the tree-ring formation period had ended; hence, δ^18^O_sw_ featured an increasing trend during the tree-ring formation period (Figure 3b, lines with markers). Värriö was further characterized by lower δ^18^O_sw_ except for 2010 and 2018, when δ^18^O_sw_ at both sites aligned. This was caused by precipitation δ^18^O, which, on average, was 2.3‰ lower at Värriö than at Hyytiälä. In general, δ^18^O_ring_ values were also lower in Värriö than in Hyytiälä except for 2017 (Figure 3c). In terms of seasonal patterns, δ^18^O_ring_ differed between the two sites but was generally more stable over the growing season compared with the variations observed in δ^18^O_sw_ and RH. In addition, Figure 3c shows the variability between trees (semitransparent lines), which was larger for Värriö (mean SD between trees 0.97‰) than for Hyytiälä (SD = 0.66‰). Also, correlations between trees were weaker in Värriö (*r* = 0.00–0.36) compared with Hyytiälä (*r* = 0.21–0.59). For comparison, alignment between trees for δ^13^C_ring_ was characterized by SD = 0.68 and *r* = 0.60–0.81 in Hyytiälä and SD = 0.59 and *r* = 0.50–0.90 in Värriö, indicating much more consistent behavior between trees in δ^13^C.

**Figure 4:**
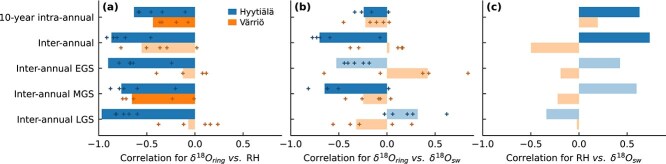
Pearson correlation coefficients for Hyytiälä and Värriö showing the relationship between (a) δ^18^O of tree rings (δ^18^O_ring_) and relative humidity (RH), (b) δ^18^O_ring_ and δ^18^O of source water (δ^18^O_sw_) and (c) RH and δ^18^O_sw_. Correlations are presented for various temporal scales (*y*-axis): whole timeseries (10-year intra-annual), data averaged by year (inter-annual) and yearly averaged data grouped by early growing season (interannual EGS), mid-growing season (interannual MGS) and late growing season (interannual LGS). Bars represent correlations computed for data averaged over the measured trees (nonsignificant correlations, *P* > 0.05, are shown as semi-transparent bars), and markers indicate correlations for individual trees.

The correlations between δ^18^O_ring_, RH and δ^18^O_sw_ showed different patterns at the two sites, over different temporal scales and among individual trees within the same site (Figure 4). Irrespective of temporal scales, the correlations between δ^18^O_ring_ and RH (Figure 4a) were typically stronger than the correlation with δ^18^O_sw_ within a site (Figure 4b). At Hyytiälä, we found statistically significant correlations (*P* < 0.05) between RH and δ^18^O_ring_ for all investigated temporal scales (−0.97 to −0.63). The negative correlation between δ^18^O_ring_ and RH aligns with expectations, as the Craig–Gordon model predicts increase of leaf water δ^18^O with decreasing RH due to evaporative enrichment. If the variability of δ^18^O_ring_ were driven mainly by δ^18^O_sw_, a positive correlation between δ^18^O_ring_ and δ^18^O_sw_ would be expected (Eq. (8)). However, at Hyytiälä, these correlations were mostly negative (Figure 4b). This was caused by δ^18^O_sw_ at Hyytiälä consistently correlating negatively with RH (Figure 4c), except for the late growing season (LGS), where correlations between both δ^18^O_ring_ and RH against δ^18^O_sw_ become positive. Late growing season also had the most negative RH correlation at Hyytiälä. At Värriö, correlations between δ^18^O_ring_ and RH were weaker and less consistent among trees compared with Hyytiälä (Figure 4a). Still, they were mostly negative, as expected, with the strongest correlation for mid-growing season (MGS; −0.65). At Värriö, neither the correlations between δ^18^O_sw_ and δ^18^O_ring_ nor those between δ^18^O_sw_ and RH were statistically significant (Figure 4b and c).

### Modeled tree-ring δ^18^O

**Figure 5:**
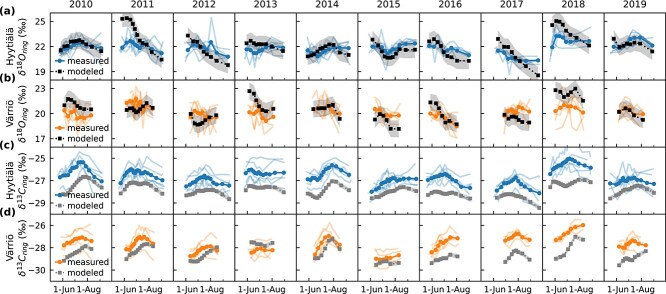
Comparison between modeled and measured intra-annual tree-ring δ^18^O (δ^18^O_ring_) at (a) Hyytiälä and (b) Värriö, and δ^13^C_ring_ at (c) Hyytiälä and (d) Värriö. Semitransparent lines represent measured isotope series of individual trees, and lines with markers denote isotope series averaged over the trees. Gray-shaded areas around modeled series indicate uncertainty related to varying lignin fraction.

The model replicated the observed intra-annual δ^18^O_ring_ (Figure 5a and b), with MAEs of 0.77‰ for Hyytiälä and 0.90‰ for Värriö. The model captured the seasonal δ^18^O_ring_ patterns better at Hyytiälä than at Värriö, which was expected based on the stronger correlation between RH and δ^18^O_ring_ at Hyytiälä (Figure 4a). However, at Hyytiälä, the model tended to overestimate δ^18^O_ring_ during the early growing season and underestimate it during the late growing season for most years. This pattern could plausibly be explained by a rising lignin fraction during the growing season (gray-shaded area in Figure 5a), which has a much larger impact on δ^18^O_ring_ than δ^13^C_ring_ (Figure 5c). At Värriö, there was no clear systematic seasonal under- or overestimation; seasonal patterns were only captured during some individual years (e.g., 2014 and 2019; Figure 5b). The correlation between model and measured intra-annual δ^18^O_ring_ was 0.66 (*P* < 0.01) at Hyytiälä and 0.32 (*P* < 0.01) at Värriö. For δ^13^C, the model performed better in capturing intra-annual patterns (Figure 5c and d): correlations between measured and modeled intra-annual δ^13^C_ring_ were 0.82 (*P* < 0.01) at Hyytiälä and 0.51 (*P* < 0.01) at Värriö. However, there was a rather consistent offset, especially at Hyytiälä, between measured and modeled intra-annual δ^13^C_ring_, resulting in MAE 1.0‰ and 1.32‰ at Hyytiälä and Värriö, respectively. The offset may be caused by missing postphotosynthetic processes (e and x in Eq. (7) could not resolve the offset) or neglected variation of leaf sugar δ^13^C between different needle ages and canopy positions. However, as modeling of δ^13^C_ring_ was only included in this study to provide additional constraint on the time integration aspects, we did not investigate this further.

**Figure 6:**
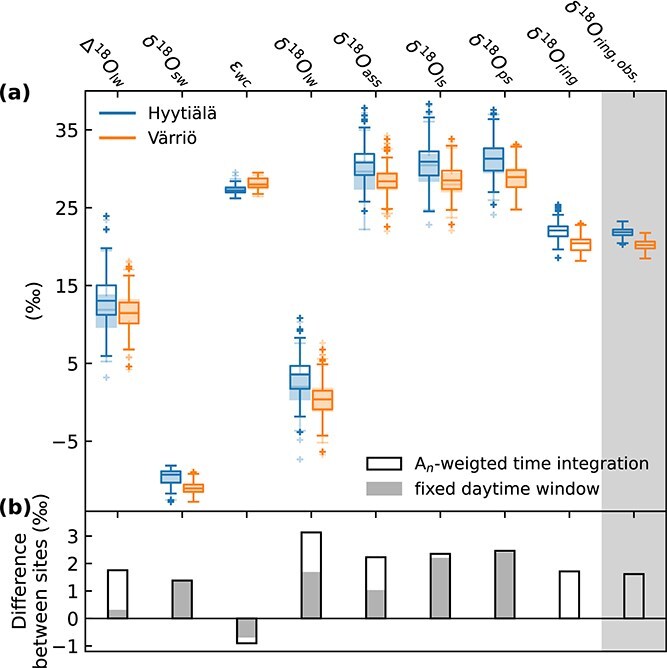
(a) Distributions of model outputs along the pathway from leaf to tree rings (Figure 2) and observed distributions of mean tree-ring δ^18^O (δ^18^O_ring,obs._, gray-shaded area) at Hyytiälä and Värriö 2010–19, and (b) the difference in mean values between the two sites. Model outputs include evaporative enrichment (Δ^18^O_lw_), δ^18^O of source water (δ^18^O_sw_), temperature-dependent biochemical fractionation factor (*ε*_wc_), δ^18^O of leaf water (δ^18^O_lw_), δ^18^O of new assimilates (δ^18^O_ass_), δ^18^O of sugar in the leaf pool (δ^18^O_ls_), δ^18^O of sugar in the phloem pool (δ^18^O_ps_) and tree-ring δ^18^O (δ^18^O_ring_). Distributions comprise values corresponding to growth time windows of tree-ring subsections to be comparable with δ^18^O_ring_. Boxplots (a) and bars (b) with outlines include data weighted by modeled net CO_2_ exchange (*A*_n_), while shaded filled boxplots and bars show data restricted to 9 a.m. to 3 p.m. for comparison.

As shown already in Figure 3c, observed δ^18^O_ring_ values were higher in Hyytiälä compared with Värriö. The observed mean difference was 1.6‰, which was similar to the modeled difference of 1.7‰. To analyze the cause of this difference, Figure 6a shows the distributions of model outputs for both sites side by side along the pathway from leaf to tree rings (Figure 2), as well as the distribution of observed δ^18^O_ring_ for comparison. Figure 6 shows that the difference in evaporative enrichment (Δ^18^O_lw_) between the two sites was the main cause for difference in δ^18^O_ring_, while the difference in δ^18^O_sw_ was partly canceled by the opposite difference in temperature-dependent *ε*_wc_. The difference between the two sites in new assimilates (δ^18^O_ass_) was thus not much larger than that of Δ^18^O_lw_ (Figure 6b), as δ^18^O_ass_ ≈ δ^18^O_sw_ + Δ^18^O_lw_ + *ε*_wc_. While no fractionation occurs in the model as new assimilates travel through leaf and phloem sugar pools (Figure 2), the differences between the two sites remained rather stable (Figure 6b). Finally, the difference between the two sites decreases once reaching δ^18^O_ring_ (Figure 6b) as oxygen atom exchange with source water during cellulose synthesis dampens the remaining difference (Figure 2), i.e., δ^18^O_ring_ ≈ *p*_ex_(δ^18^O_sw_ + *ε*_wc_) + (1 − *p*_ex_) δ^18^O_ps_ − 4.4‰ (offset between cellulose and resin-extracted wood δ^18^O). Interestingly, we found that if leaf-level differences were to be calculated using a constant daytime period, including data from 9 a.m. to 3 p.m., the difference in evaporative enrichment between the two sites would almost disappear (Δ^18^O_lw_ in Figure 6b).

**Figure 7:**
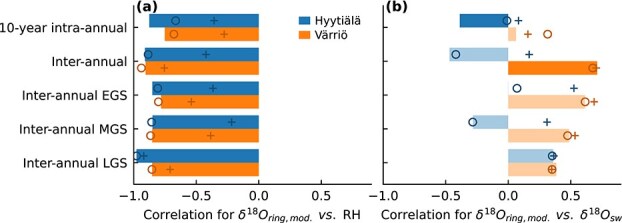
Pearson correlation coefficients for Hyytiälä and Värriö showing the relationship between (a) modeled δ^18^O of tree rings (δ^18^O_ring,mod._) and relative humidity (RH) and (b) δ^18^O_ring,mod._ and δ^18^O of source water (δ^18^O_sw_). Correlations are presented for various temporal scales (*y*-axis): whole timeseries (10-year intra-annual), data averaged by year (inter-annual), and yearly averaged data grouped by early growing season (interannual EGS), mid-growing season (interannual MGS) and late growing season (interannual LGS). Bars represent correlations for model results with constant *p*_ex_ as presented in section Modeled tree-ring δ^18^O (nonsignificant correlations, *P* > 0.05, are shown as lighter-colored bars), and markers represent correlations for model results with varying apparent *p*_ex_ (section Sensitivity to varying p_ex_) based on day of the year (circles; Figure 9a) and RH (plus signs; Figure 9b).

The modeled δ^18^O_ring_ values correlated more strongly with RH than with δ^18^O_sw_ (Figure 7) as was observed for the actual data (Figure 4). However, the correlation of modeled δ^18^O_ring_ with RH at intra-annual resolution and for Värriö were much stronger (Figure 7a) than that observed in the data (Figure 4a). This may be due to, e.g., uncertainties underlying the data analysis (namely, pairing intra-annual tree-ring subsections with corresponding growth time windows) or missing model processes (e.g., varying *p*_ex_) that interfere with the intra-annual RH signal in δ^18^O_ring_.

### Sensitivity to dating and time integration

**Figure 8:**
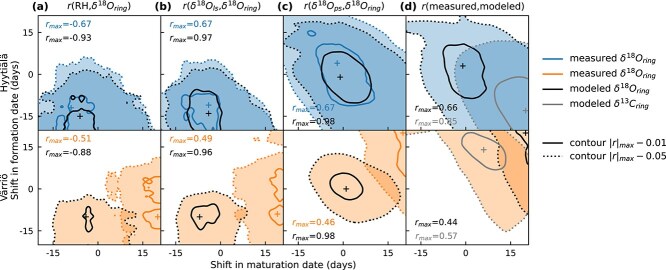
Sensitivity analysis of formation and maturation dates assigned by xylogenesis to correlations of modeled and measured intra-annual tree-ring δ^18^O (δ^18^O_ring_) against (a) RH, (b) modeled leaf sugar δ^18^O (δ^18^O_ls_) and (c) modeled phloem sugar δ^18^O (δ^18^O_ps_). Additionally, panel (d) shows correlations of measured and modeled δ^18^O_ring_ and δ^13^C_ring_. Pearson correlations (r) were calculated across temporal windows modified by moving formation and maturation dates assigned xylogenesis by ±20 days. Results are shown for the two study sites, Hyytiälä and Värriö. Markers indicate peak correlation values (|r|_max_), contours are shown for |*r*|_max_ − 0.01 (solid lines) and |*r*|_max_ − 0.05 (dotted line). RH, δ^18^O_ls_ and δ^18^O_ps_ were weighted based on modeled net CO_2_ exchange (*A*_n_) within time windows.

We conducted a sensitivity analysis to investigate whether misalignments in time-integration (leaf and phloem pools; Figure 2) or timing and duration of wood formation (assigned by xylogenesis; Figure 1b and c) could explain the differences between modeled and measured RH correlations at intra-annual resolution. This approach involved examining correlations of modeled and measured intra-annual δ^18^O_ring_ with RH and modeled sugar δ^18^O values across temporal windows modified by moving formation and maturation dates by ±20 days (Figure 8a–c). Additionally, we examined whether the correlation between modeled and measured tree-ring isotopes indicated any systematic uncertainty related to the time windows assigned by xylogenesis (Figure 8d).

For Hyytiälä, results were similar for modeled and measured δ^18^O_ring_, i.e., correlations peaked at similar offsets from the center point (Figure 8a–c). Correlations with RH peaked approximately when the formation date was shifted by ~10–15 days and the maturation date by 5–10 days prior to the dates assigned by xylogenesis. However, the effect on the strength of the data-based correlation between δ^18^O_ring_ and RH was modest, from −0.63 to −0.67 (Figure 8a). Still, this adjustment does not resolve the discrepancy in intra-annual RH correlation strength between measured and modeled data. According to the model, a ~10-day delay results from integration and mixing processes in the leaf and, especially, in the phloem (Figure 2), as shown by the gradual shift of the peak correlation toward the center point from Figure 8a to Figure 8c. In line with this, Figure 8d suggests no improvement in δ^18^O_ring_ model fit was achieved by modifying time windows. Contrarily, the δ^13^C_ring_ model fit would slightly benefit from extended time windows with earlier formation dates and delayed maturation dates.

At Värriö, peak correlation values did not align between modeled and measured as they did at Hyytiälä (Figure 8a–c). While peak correlations for modeled δ^18^O_ring_ expectedly occurred at similar offsets from the dates assigned by xylogenesis as at Hyytiälä due to identical parametrization, measured δ^18^O_ring_ correlated most strongly with RH, leaf sugar δ^18^O and phloem sugar δ^18^O with clearly modified time windows (Figure 8a–c). For RH and leaf sugar δ^18^O, a 15–20-day delay in maturation dates was suggested (Figure 8a and b), and for phloem sugar δ^18^O, a delay in both formation and maturation dates of up to 20 days was needed to obtain the highest correlation with measured δ^18^O_ring_ (Figure 8c). Similarly, delays from dates assigned by xylogenesis were evident from Figure 8d, where the fit of both δ^18^O_ring_ and δ^13^C_ring_ models improved toward the upper-right corner (for δ^18^O_ring_ from 0.32 to 0.44 and for δ^13^C_ring_ from 0.51 to 0.57).

The sensitivity of model fit to assigned time windows by xylogenesis (as in Figure 8d) was also explored on a yearly basis (Figure S3 available as Supplementary Data at *Tree Physiology* Online). This revealed that at Hyytiälä, there were only some years (2011 and 2015), when shifts in time windows consistent between δ^18^O_ring_ and δ^13^C_ring_ models would improve their fit. At Värriö, this was more common with improvement to both models’ fit with adjustments to time windows in the years 2013, 2014, 2016 and 2018.

### Sensitivity to varying apparent p_ex_

**Figure 9:**
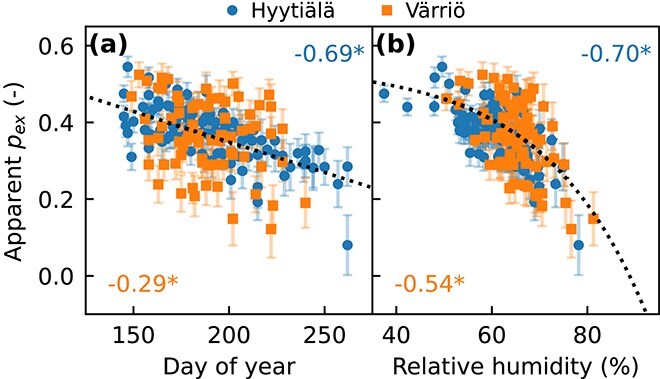
Relationship between apparent *p*_ex_ and (a) day of the year and (b) relative humidity (RH) for Hyytiälä and Värriö over 2010–19. Apparent *p*_ex_ was calculated using observed tree-ring δ^18^O, and modeled phloem sugar and source water δ^18^O (Eq. (8)). Pearson correlations are shown in the upper-right corner for Hyytiälä and the lower-left corner for Värriö. Asterisks denote statistical significance (*P* < 0.05). Dotted lines show regression functions fitted to the dataset pooled over both sites to be used in alternative model simulations with varying apparent *p*_ex_. Error bars indicate uncertainty in apparent *p*_ex_ corresponding to ±0.5‰ uncertainty in observed tree-ring δ^18^O.

Next, we explored whether varying *p*_ex_ could explain the differences between the intra-annual RH signal in measured and modeled δ^18^O_ring_. For this purpose, *p*_ex_ corresponding to each tree-ring subsection was solved from Eq. (8), using measured δ^18^O_ring_ (*R*_cel,i_ in Eq. (8) after correction for offset between wood and cellulose, 4.4‰) and modeled contributions of δ^18^O in phloem sugar and source water (integral terms in Eq. (8)). We call this apparent *p*_ex_ (*p*_ex′_), as there is no guarantee that all exchange is happening during cellulose synthesis. The resulting *p*_ex′_ showed a statistically significant correlation with both day of the year (DOY) and RH at both sites (Figure 9). To investigate how potentially varying *p*_ex′_ influences the RH-signal preserved in δ^18^O_ring_, we modeled δ^18^O_ring_ (Eq. (8)) using two alternative approaches: (i) *p*_ex′_ defined as a linear function of DOY (Figures 9a and S4 available as Supplementary Data at *Tree Physiology* Online) and (ii) *p*_ex′_ defined as a nonlinear function of RH (Figures 9b and S5 available as Supplementary Data at *Tree Physiology* Online). Both functions were fitted to the dataset pooled over the two sites. When varying *p*_ex′_ was accounted for, the intra-annual correlations between modeled δ^18^O_ring_ and RH weakened (Figure 7a). With the DOY-based *p*_ex′_, the intra-annual correlation at Hyytiälä was −0.66, very close to the observation-based value of −0.63. At interannual resolution, the DOY-based *p*_ex′_ had close to no effect: correlations at Hyytiälä remained strong (−0.98 to −0.81) and comparable to the observation-based range (−0.97 to −0.76). Contrary, RH-based *p*_ex′_ weakened also the intra-annual correlations (Figure 7a). With the DOY-based *p*_ex′_, the model fit for Hyytiälä improved from *r* = 0.66 to *r* = 0.73 and the MAE from 0.77‰ to 0.57‰. For Värriö, interpreting the results was more challenging as the model did not capture the observed δ^18^O_ring_ variability well to begin with.

## Discussion

### Dominance of relative humidity over source water δ^18^O

**Figure 10:**
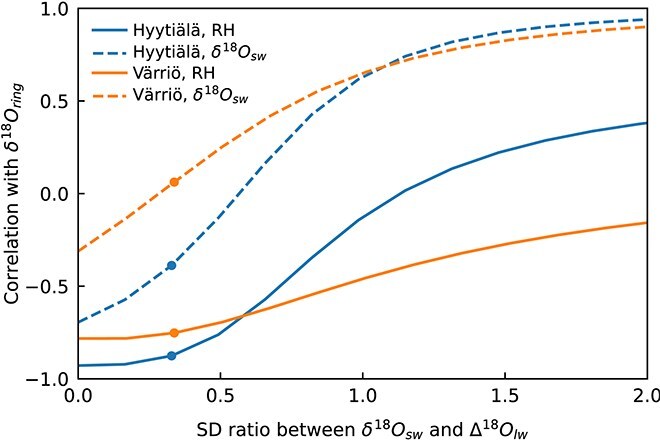
Changes in correlations of modeled intra-annual tree-ring δ^18^O (δ^18^O_ring_) with relative humidity (RH) and δ^18^O of source water (δ^18^O_sw_), when their variability relative to one and the other was changed (*x*-axis, quantified as the ratio between the SD of δ^18^O_sw_ and the SD of leaf water evaporative enrichment (Δ^18^O_lw_)). The results were obtained with model scenario runs, where the variability of the model input δ^18^O_sw_ was, in turn, decreased and increased around its mean value. Scenarios were run for both sites and markers indicate the actual conditions at the sites (i.e., correlations corresponding to 10-year intra-annual correlations in Figure 7).

The RH signal originating from leaf-level evaporative enrichment dominated the variation of δ^18^O_ring_ over that of δ^18^O_sw_ at both sites (Figures 4 and 7), in line with earlier studies (Wernicke et al. 2015, Szymczak et al. 2019, Xu et al. 2022). The dominance of RH was caused by its higher relative variability compared with δ^18^O_sw_. This can be quantified from Figure 6, where modeled evaporative enrichment (Δ^18^O_lw_), which represents variation mainly driven by RH (Cernusak et al. 2022), was ~3.5 times higher than the variation of δ^18^O_sw_ (in terms of SD and at the time resolution corresponding to tree-ring subsections). If the ratio between the SD of Δ^18^O_lw_ and the SD of δ^18^O_sw_ were to change, for example, due to changes in temporal or spatial scales, the dominant environmental signal shaping δ^18^O_ring_ would also shift. Figure 10 illustrates correlations of modeled δ^18^O_ring_ with RH and δ^18^O_sw_ obtained over a range scenario runs with varying variability in δ^18^O_sw_ vs Δ^18^O_lw_, explaining why RH signals are not always found in δ^18^O_ring_ (Treydte et al. 2014, Fan et al. 2021, Wang et al. 2021). However, the relative variability of RH and δ^18^O_sw_ is not the only thing that matters but also their correlation with one another (Figure 4c), which causes the different behavior between Hyytiälä (negative correlation between RH and δ^18^O_sw_) and Värriö (no correlation between RH and δ^18^O_sw_) in Figure 10.

Despite a rather poor model fit at Värriö, the model still captured the overall level of δ^18^O_ring_ and the mean difference between Värriö and Hyytiälä well. Complementing our dataset with modeling, we were able to identify that the difference in δ^18^O_ring_ between the two sites was also mainly driven by RH, i.e., differences in evaporative enrichment (Figure 6; Li et al. 2015, Watanabe et al. 2023). This became evident only when the analysis (Figure 6) was done with values weighted by modeled net CO_2_ exchange (*A*_n_) opposed to a fixed daytime window (e.g., 9 a.m. to 3 p.m.). The latter ignores the latitudinal difference in photosynthetically active hours between the sites, while the former corresponds more closely to the aggregation in the model (Figure 2). Identifying the underlying causes for differences in δ^18^O_ring_ solely based on empirical data can be challenging as samples are typically taken only at a certain time of the day, representing instant (δ^18^O_lw_) or previous days to week (δ^18^O_ls_ and δ^18^O_ps_) environmental conditions (Treydte et al. 2014, Martínez-Sancho et al. 2023, Szejner et al. 2025).

Strongest correlations with RH were obtained for interannual resolution, and for Hyytiälä, all interannual-based correlations were stronger than the one calculated from the full 10-year intra-annual dataset (Figure 4a). Then again, model results showed strong RH signals across inter- and intra-annual resolutions (Figure 7a), which suggested that possible opposing trends in RH and δ^18^O_sw_ signals were not causing the weakened RH signal obtained from our 10-year intra-annual data. However, for individual years, opposing patterns in RH and δ^18^O_sw_ signals were likely relevant, as suggested by Szejner et al. (2025). They studied the intensively monitored years 2018–19 in the same sites but could not detect an RH signal in δ^18^O_ring_, despite its presence in δ^18^O_lw_ and δ^18^O_ls_. Our modeling results supported this, as correlations between modeled intra-annual δ^18^O_ring_ and RH for individual years ranged from −0.97 to 0.10. To conclude, the interpretation of 10-year-long intra-annual signals in our dataset was hindered by other factors, such as time alignment of tree-ring subsections, integration of data over selected time windows or seasonal processes such as varying lignin fraction or *p*_ex_.

### Time alignment and integration complicate interpretation of intra-annual tree-ring δ^18^O

The interpretation of intra-annual tree-ring isotopic values requires information on timing and duration of wood formation to pair the analyzed tree-ring subsections with time windows (Walcroft et al. 1997, Ogée et al. 2009, Pérez-de-Lis et al. 2022). These can be derived from direct measurements, such as micro-core sampling (Rinne et al. 2015, Martínez-Sancho et al. 2023), but this restricts the analysis to sites with intensive monitoring and typically a limited number of years. The benefit of stable isotope values in tree rings is that they provide archives extending beyond intensive monitoring periods or locations (McCarroll and Loader 2004, Gessler et al. 2014). Thus, despite the potentially added uncertainty, reliance on growth models (Schiestl-Aalto et al. 2015, Friend et al. 2022) in facilitating the time alignment of tree-ring subsections is imperative. We applied the CASSIA model, well established for boreal conifers (Schiestl-Aalto et al. 2015), to derive the start of tracheid formation and end of maturation for each tree-ring subsection. These time windows were used to derive corresponding environmental conditions and model intra-annual δ^18^O_ring_. Based on a sensitivity analysis on dates assigned by the CASSIA model, we were not able to reveal any systematic misalignment in the assigned time windows for Hyytiälä (Figure 8). It is worth noting that the strongest correlation of both modeled and measured δ^18^O_ring_ with RH was not obtained with the time window assigned by xylogenesis, but a time window offset by about −10 days, which corresponds to the mean travel time of new assimilates through leaf and phloem sugar pools to tree rings in the model (Figure 2). Similar travel times have been reported earlier (Gessler et al. 2009, Rog et al. 2021), but such offsets are seldom accounted for in the interpretation of intra-annual tree-ring isotopic data (Martínez-Sancho et al. 2023). Another aspect of time integration is the weighting of hours within days and days along the assigned time window when comparing RH and δ^18^O_sw_ to δ^18^O_ring_. Commonly, no filtering or a set daytime window is used (Szymczak et al. 2019, Szejner et al. 2025), but our results suggest that weighting based on net CO_2_ exchange (*A*_n_) is more justified (Cernusak et al. 2005, Leppä et al. 2022). For Hyytiälä, the *A*_n_-based weighting compared with a set daytime window (9 a.m. to 3 p.m.) strengthened the correlations of intra-annual δ^18^O_ring_ with RH from −0.58 to −0.63. Notably, a −0.63 correlation coefficient was also obtained using weighting based on clear-sky global radiation, which has a wider applicability as it is not dependent on meteorological data or extensive modeling.

Modeling the δ^18^O_ring_ signals at Värriö was more challenging than at Hyytiälä. However, the model was able to predict δ^18^O values on the pathway to tree rings (i.e., leaf water, leaf and phloem WSC) with close to similar accuracy as for Hyytiälä (Figure S2c–e) available as Supplementary Data at *Tree Physiology* Online; hence, we expect the uncertainties to lie in wood formation processes, including influences of δ^18^O_sw_. Higher variation in δ^18^O_ring_ between trees at Värriö compared with Hyytiälä may also point to these factors being more variable between trees at Värriö and challenge the attempt to model an average tree. For δ^13^C_ring_, the behavior between trees was much more consistent within sites and between-tree correlations were of similar magnitude at both sites. Still, also for δ^13^C_ring_, the model performed worse at Värriö, suggesting dating and time integration caused more uncertainties at Värriö, which was also supported by our sensitivity analysis on dates assigned by xylogenesis (Figure 8d). Additionally, the generally worse performance of modeled δ^18^O_ring_ (0.32–0.66) compared with δ^13^C_ring_ (0.51–0.82) may be related to potentially varying lignin fraction (which δ^13^C_ring_ is almost unaffected by; Figure 5), simplifications in δ^18^O_sw_ modeling (especially at Värriö; Figure S2b available as Supplementary Data at *Tree Physiology* Online) or missing processes, such as varying fraction of oxygen exchange. In northern latitudes such as Värriö, δ^18^O_sw_ is likely strongly influenced by meltwater and may not reflect soil water δ^18^O during the growing season (Muhic et al. 2023), which is neglected in our modeling approach. Overall model performance, especially for δ^18^O_ring_, was rather modest, but it was comparable or superior to limited earlier studies modeling intra-annual isotopic signals in tree rings (Ogée et al. 2009, Zeng et al. 2017).

The Hyytiälä site showed that growth models provide a promising tool for the time alignment of thin tree-ring subsections (Tang et al. 2023), despite the misalignment between modeled and micro core-based growth curves (Figure S1 available as Supplementary Data at *Tree Physiology* Online). Applying dendrometer data alongside micro coring data, whose accuracy suffers from its destructive nature, could provide additional constraints to the modeled growth curves. Still, dating based on growth models provides benefits over simple monthly averaging approaches (Szymczak et al. 2019, Watanabe et al. 2023) and advances process-based modeling of intra-annual isotopic signals (Ogée et al. 2009). This enables the interpretation and unprecedented modeling of decadal records of intra-annual δ^18^O_ring_, an analysis that has recently seen marked advancement with the establishment of a laser ablation protocol for δ^18^O (Sahlstedt et al. 2025). Nevertheless, it is doubtful that growth models and time integration over obtained growth time windows can reach such accuracy that data-based intra-annual correlations would compare with correlations calculated for model outcomes (Figure 4 vs Figure 7). Correlation analysis of model outcomes does not suffer from these uncertainties, as the same time windows assigned for the tree-ring subsections are used in the model and for calculating corresponding environmental data. Thus, despite no systematic mismatch in time alignment for Hyytiälä being found (Figure 8), the weakened intra-annual RH signal was still likely, at least partly, due to uncertainties in time alignment and integration.

### Mystery of oxygen exchange with surrounding water

Among the processes controlling δ^18^O along the pathway from source water to the tree rings, oxygen exchange and its variability on the route from leaves to tree-ring cellulose is one of the least understood factors (Song et al. 2022). Conventionally, oxygen exchange during cellulose synthesis (*p*_ex_) is assumed rather invariable, with a value close to 0.4 (Sternberg et al. 1986, Roden et al. 2000, Cernusak et al. 2005). However, recent studies suggest that *p*_ex_ may vary with environmental conditions and physiology (Gessler et al. 2013, Song et al. 2014, Cheesman and Cernusak 2017, Szejner et al. 2020). For example, longer turnover times of the sugar and starch pools may increase the likelihood of triose-phosphate cycling, exposing more carbonyl oxygen to exchange with xylem water and leading to higher *p*_ex_ values (Song et al. 2014). Additionally, but following this same mechanism, *p*_ex_ may vary across species and along environmental gradients, such as aridity. Increased aridity can lead to higher *p*_ex_ due to changes in metabolic activity and water availability (Cheesman and Cernusak 2017, Holloway-Phillips et al. 2023, Martínez-Sancho et al. 2023). Albeit typically defined as oxygen exchange during cellulose synthesis, *p*_ex_ is commonly calculated using leaf water δ^18^O (Belmecheri et al. 2018, Szejner et al. 2020, Martínez-Sancho et al. 2023) and therefore, it remains unclear where the variable oxygen exchange happens. In line with recent studies (Fiorella et al. 2022, Pan et al. 2024), our modeling suggested oxygen exchange was already happening during phloem loading and transport (Figure S2e available as Supplementary Data at *Tree Physiology* Online). Hereby, we report variability in apparent *p*_ex_ (*p*_ex′_) without specifying where the variable oxygen exchange takes place. Our results (Figure 9) suggested that *p*_ex′_ decreases along the growing season (Offermann et al. 2011) and with increasing RH (Martínez-Sancho et al. 2023). Based on model runs, we show that time-varying *p*_ex′_ adds complexity to the interpretation of δ^18^O_ring_ as it interferes with the environmental signals, especially at intra-annual resolution (Figure 7).

Despite the potential explanation that varying *p*_ex′_ provides to the lower RH signal of δ^18^O_ring_ at intra-annual resolution, we hesitate to treat this pattern as evidence that *p*_ex′_ definitively is variable. First, δ^18^O_ring_ was analyzed from resin-extracted wood, not cellulose. An increase in lignin fraction during the growing season provides an alternative explanation for the mismatch in intra-annual δ^18^O_ring_ patterns (Figure 5a and b). However, an increase in lignin fraction from earlywood to latewood is not supported in the literature (Fredriksson et al. 2018). Changes in the δ^18^O offset between lignin and cellulose during the growing season could potentially also explain the observed mismatch. In retrospect, parallel analysis of a subset of cellulose-extracted samples would have been critical to resolve this. Second, exactly opposite seasonal trends (Szejner et al. 2020, Martínez-Sancho et al. 2023) and relationships with RH at the leaf level (Lehmann et al. 2016, Hirl et al. 2021) compared with our results have also been reported in addition to those that are in line with our results (Offermann et al. 2011, Martínez-Sancho et al. 2023). Using model results, we were also able to show that applying the suggested weighting by *A*_n_ was necessary to decrease uncertainty in estimated *p*_ex′_, when calculating *p*_ex′_ from leaf sugar δ^18^O or leaf water δ^18^O (Figure S6 available as Supplementary Data at *Tree Physiology* Online; Martínez-Sancho et al. 2023, Szejner et al. 2025). This is critical for studying the variability of *p*_ex′_, and uncertainties related to the estimation of *p*_ex′_ should be addressed more transparently in future studies.

Our findings, along with recent literature, suggest that more efforts are needed to explore potential variations in the proportion of oxygen exchange, associated mechanisms, where they take place, and their incorporation into models to improve interpretations of intra-annual signals in δ^18^O_ring_ (Holloway-Phillips et al. 2023, Szejner et al. 2025). Position-specific oxygen isotope analysis (Ellsworth et al. 2013, Ma et al. 2020, Dion-Kirschner et al. 2026) offers promising avenues to better understand the variation in apparent *p*_ex_. It aims to analyze individual oxygen atom positions in glucose, the building block of cellulose, which go through exchange with surrounding water at different rates, some remaining completely unexchanged (Waterhouse et al. 2013). Combined with isotopic modeling to address the complexities of time integration, position-specific isotope analysis can help resolve the relative fractions (apparent *p*_ex_) of leaf-level evaporative enrichment and source water signals in δ^18^O_ring_.

Overall, this study proved that combining data analysis and process-based modeling enabled more in-depth analysis than either approach alone. Such combined analysis bridges the gap between empirical studies, focused on the climatic signals of δ^18^O_ring_ records, and mechanistic modeling studies, which can address the underlying mechanisms resulting in climatic signals or the lack of them, advancing the interpretation of δ^18^O_ring_ records across temporal and spatial scales.

## Supplementary Material

Supplementary_materials_tpag026

## Data Availability

The model code (δ^18^O and δ^13^C) was written in Python and available at https://github.com/LukeEcomod/Leaf-to-Tree-ring-Isotopes with an example run corresponding to this study. The tree-ring δ^18^O and δ^13^C data are available through the same link. Environmental data for the study sites (SMEAR II Hyytiälä forest; SMEAR I Värriö forest) can be obtained from https://smear.avaa.csc.fi/ or https://etsin.fairdata.fi/ (Aalto et al. 2023, 2024). Other isotopic data used in the study (Figure S2 available as Supplementary data at *Tree Physiology* Online) are available upon request to the corresponding author.
