# Supplementary material for: Interpreting inter- and intra-annual environmental signals in tree-ring δ18O using isotope-enabled modeling
Source: Tree Physiol. 2026 Feb 20;46(4):tpag026. doi: 10.1093/treephys/tpag026 (PMC13064660; doi:10.1093/treephys/tpag026)
Supplement: Supplementary_materials_tpag026 [file supplementary_materials_tpag026.pdf]

Supplementary data for "Interpreting inter- and intra-annual environmental signals in tree-ring $\delta^{18}O$ using isotope-enabled modeling" by Leppä et al.

The following Supplementary data is available for this article:

Figure S1. Comparison of modeled and measured xylogenesis data.
Figure S2. Comparison of modeled and measured precipitation $\delta^{18}O$, source water $\delta^{18}O$, leaf water $\delta^{18}O$, and leaf water-soluble carbohydrates (WSC) $\delta^{18}O$ and phloem WSC $\delta^{18}O$ during growing seasons 2018–2019.
Figure S3. Yearly based sensitivity analysis of $\delta^{18}O$ and $\delta^{13}C$ model fit to time windows assigned by xylogenesis.
Figure S4. Comparison of modeled and measured tree-ring $\delta^{18}O$ at Hyytiälä and Värriö with apparent p_{ex} varying based on the day of the year.
Figure S5. Comparison of modeled and measured tree-ring $\delta^{18}O$ at Hyytiälä and Värriö with apparent p_{ex} varying based on relative humidity.
Figure S6. Sensitivity of the relationship between apparent p_{ex} and day of the year or relative humidity to the calculation method.
Methods S1. Modeling source water $\delta^{18}O$
Methods S2. Effects of varying lignin content on resin-extracted wood $\delta^{18}O$ and $\delta^{13}C$

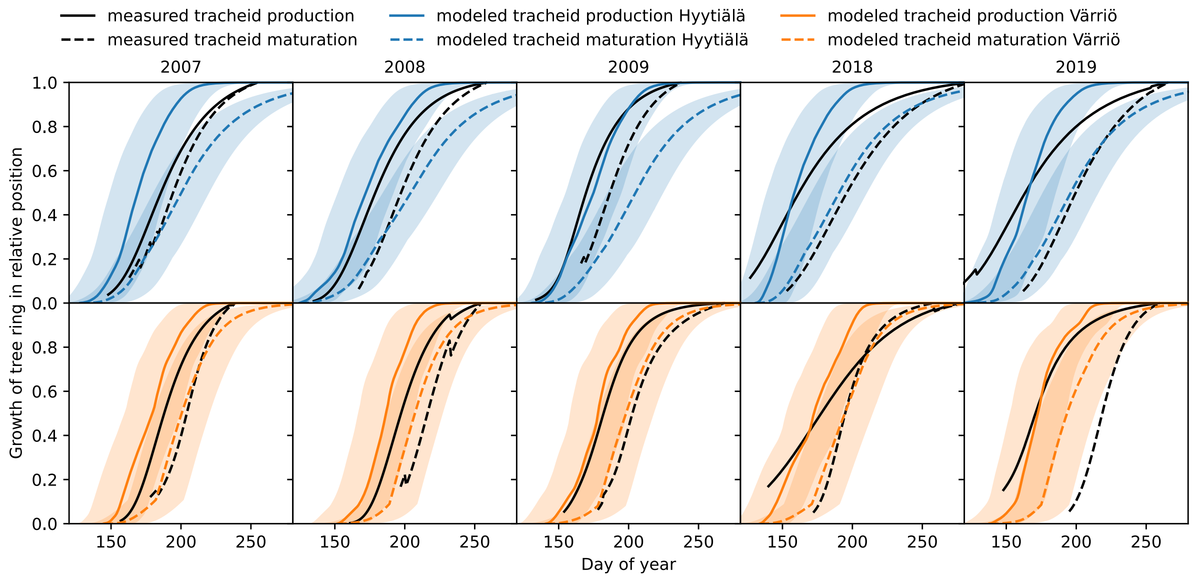

Figure S1. Comparison of modeled and measured xylogenesis data. Details on deriving growth curves from micro-core data can be found in Tang et al. (2023). Shaded areas show investigated uncertainty in modeled growth curves.

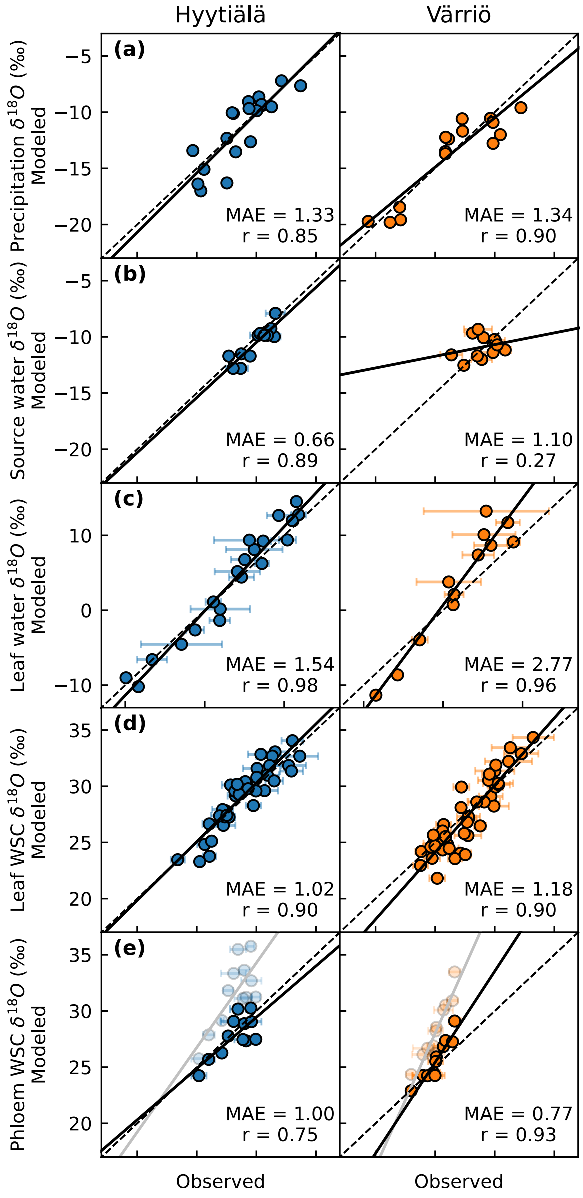

Figure S2. Comparison between modeled and measured (a) precipitation $\delta^{18}O$, (b) source water $\delta^{18}O$, (c) leaf water $\delta^{18}O$, and (d) leaf water-soluble carbohydrates (WSC) $\delta^{18}O$ and (e) phloem WSC $\delta^{18}O$ for the two sites (columns) during growing seasons 2018–2019. The modeled precipitation $\delta^{18}O$ corresponds to IsoGSM data (Yoshimura et al., 2011, 2008) that has been corrected by a site-specific offset and is compared against observations at monthly timescale (offset 2.99‰ and 3.34‰ for Hyytiälä and Värriö, respectively). Modeled $\delta^{18}O$ of phloem WSC is shown with (non-transparent) and without (semi-transparent) accounting for exchange of oxygen atoms with source water during phloem loading (Gessler et al., 2013). Pearson correaltion (r) and mean absolute error (MAE) are included for reference. Error bars indicate the SDs of the five sampled trees.

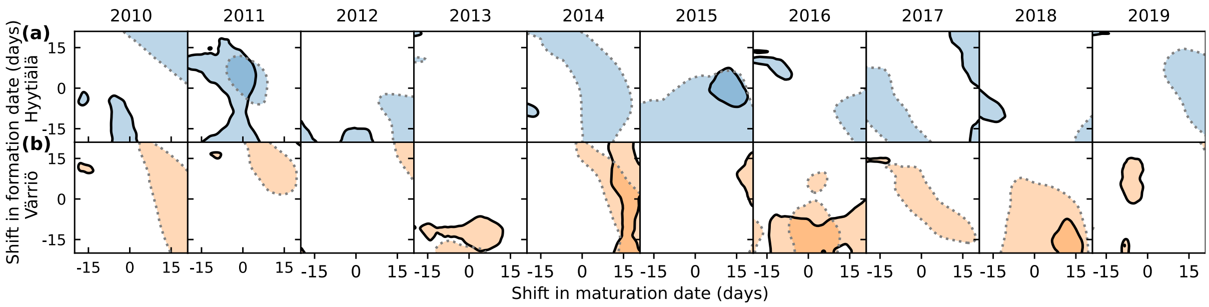

Figure S3. Sensitivity analysis of formation and maturation dates assigned by xylogenesis to correlations of measured and modeled $\delta^{18}O_{ring}$ (black solid line) and $\delta^{13}C_{ring}$ (gray dotted line) in 2010–2019. Pearson correlations (r) were calculated across temporal windows modified by moving formation and maturation dates assigned xylogenesis by ±20 days. Results are shown for the two study sites: (a) Hyytiälä and (b) Värriö. Contours are shown for peak correlation r_{max}-0.05.

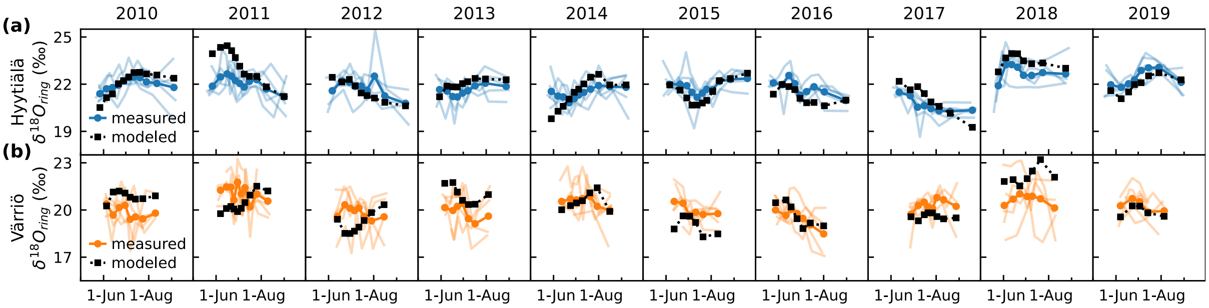

Figure S4. Comparison between modeled and measured tree-ring $\delta^{18}O$ ($\delta^{18}O_{ring}$) at (a) Hyytiälä and (b) Värriö. Model results are obtained with apparent p_{ex} varying based on the day of the year (Figure 9a). Semi-transparent lines represent measured $\delta^{18}O_{ring}$ of individual trees, and lines with markers denote $\delta^{18}O_{ring}$ series averaged over trees.

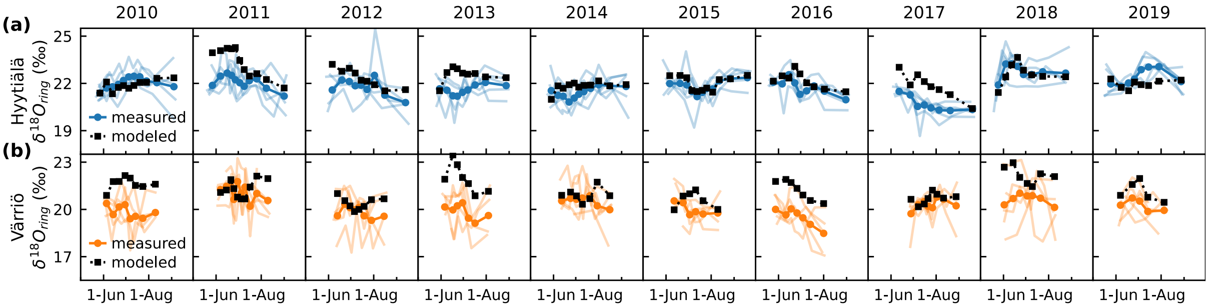

Figure S5. Comparison between modeled and measured tree-ring $\delta^{18}O$ ($\delta^{18}O_{ring}$) at (a) Hyytiälä and (b) Värriö. Model results are obtained with apparent p_{ex} varying based on relative humidity (Figure 9b). Semi-transparent lines represent measured $\delta^{18}O_{ring}$ of individual trees, and lines with markers denote $\delta^{18}O_{ring}$ series averaged over trees.

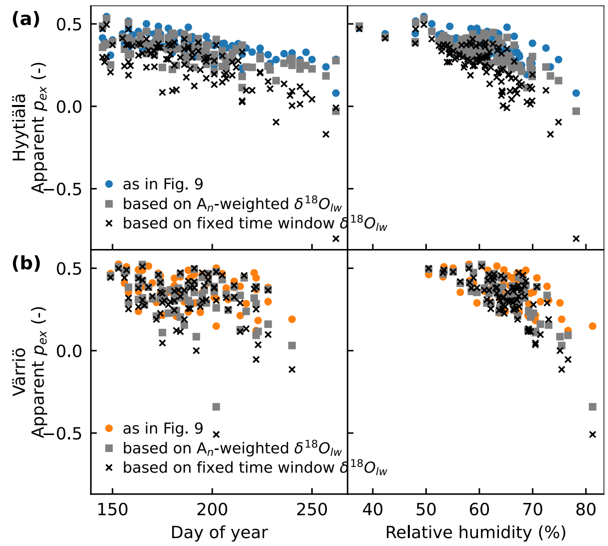

Figure S6. Comparison of apparent p_{ex} estimated as in Figure 9 and apparent p_{ex} estimated from modeled leaf water $\delta^{18}O$ ($\delta^{18}O_{lw}$) either weighted by modeled net CO_2 exchange (A_n) or using a fixed time window (9 am to 3 pm).

Methods S1. Modeling source water $\delta^{18}O$

The oxygen isotope ratio of source water (R_s) was modeled based on a mass balance approach for the soil rootzone (Leppä et al., 2022; Ogée et al., 2009):

$$\frac{dR_sW_{soil}}{dt} = R_{rain}P - R_s(ET + D) \qquad \text{(S1)}$$

where R_{rain} is the oxygen isotopic ratio of rainfall (available from IsoGSM; Yoshimura *et al.*, 2008, 2011), W_{soil} is rooting zone water storage (kg m^{-2}), and P, ET, and D are precipitation, total evapotranspiration, and drainage (kg m^{-2} d^{-1}). Eq. S1 assumes that all ET occurs without fractionation, as the forest floor at the sites is covered by a continuous moss/litter layer that blocks direct soil evaporation subject to fractionation (see also Ogée et al., 2009). We applied Eq. S1 at a daily timescale. The outflow from the rootzone (ET + D) was solved from the rootzone water budget:

$$\frac{dW_{soil}}{dt} = P - (ET + D) \qquad \text{(S2)}$$

where W_{soil} was derived from measured soil moisture assuming a rootzone depth of 0.2 m. For Värriö, soil moisture was not available for 2010, so we used the average seasonal soil moisture of the years 2011–2019.

The resulting $\delta^{18}O_{sw}$ composition was compared against the values observed for twig water in 2018–2019. The variation of observed twig water $\delta^{18}O$ was captured well in Hyytiälä (R^2=0.79) but not in Värriö (R^2=0.07); still, at both sites, the mean absolute error between modeled and measured values was reasonable (Figure S2b).

Methods S2. Effects of varying lignin content on resin-extracted wood $\delta^{18}O$ and $\delta^{13}C$

The effect of changing proportions of lignin and cellulose the isotope compositions of resin-extracted wood $\delta^{18}O$ and $\delta^{13}C$ was estimated by simple exercises using mass balance equations as demonstrated by Schleser et al. (2015):

$$\delta^{13}C_{wood} = (f_{cel}C_{cel}\delta^{13}C_{cel} + f_{lig}C_{lig}\delta^{13}C_{lig})/(f_{cel}C_{cel} + f_{lig}C_{lig}) \qquad \text{(S3)}$$

$$\Delta\delta^{13}C_{wood-cel} = \Delta\delta^{13}C_{lig-cel}(f_{lig}C_{lig})/(f_{cel}C_{cel} + f_{lig}C_{lig}) \qquad \text{(S4)}$$

where f_{cel} and f_{lig} are the cellulose and lignin content in resin-extracted wood, C_{cel} (44%) and C_{lig} (66%) are the carbon content in cellulose and lignin, and $\delta^{13}C_{wood}$, $\delta^{13}C_{cel}$ and $\delta^{13}C_{lig}$ are the isotopic compositions of resin-extracted wood, cellulose and lignin. Similarly for oxygen:

$$\delta^{18}O_{ring} = \left(f_{cel}O_{cel}\delta^{18}O_{cel} + f_{lig}O_{lig}\delta^{18}O_{lig}\right)/\left(f_{cel}O_{cel} + f_{lig}O_{lig}\right) \quad \text{(S5)}$$

$$\Delta\delta^{18}O_{wood-cel} = \Delta\delta^{18}O_{lig-cel}\left(f_{lig}O_{lig}\right)/\left(f_{cel}O_{cel} + f_{lig}O_{lig}\right) \quad \text{(S6)}$$

where O_{cel} (50%) and O_{lig} (30%) are the oxygen content in cellulose and lignin, and $\delta^{18}O_{wood}$, $\delta^{18}O_{cel}$ and $\delta^{18}O_{lig}$ are the isotopic compositions of resin-extracted wood, cellulose and lignin. Assuming a typical conifer sapwood composition with 75% of cellulose and 25% lignin (Walcroft et al., 1997) and applying the observed offsets between resin-extracted wood and cellulose ($\Delta\delta^{13}C_{wood-cel}$ = -1.1‰ and $\Delta\delta^{18}O_{wood-cel}$ = -4.4‰), we estimate the offset between lignin and cellulose to be -3.3‰ and -26.4‰ for $\delta^{13}C$ and $\delta^{18}O$, respectively. Then, allowing for the lignin fraction to vary by ±5 percentage points, we estimate the uncertainty related to lignin fraction to be ±0.2‰ and 1.0‰, for $\delta^{13}C$ and $\delta^{18}O$, respectively. While this uncertainty covers the uncertainty caused by varying lignin content, there remains additional uncertainty caused by a potentially varying offset between isotope values of cellulose and lignin (Helle et al., 2022).

References

Gessler, A., Brandes, E., Keitel, C., Boda, S., Kayler, Z.E., Granier, A., Barbour, M., Farquhar, G.D., Treydte, K., 2013. The oxygen isotope enrichment of leaf-exported assimilates – does it always reflect lamina leaf water enrichment? New Phytologist 200, 144–157. https://doi.org/10.1111/nph.12359

Helle, G., Pauly, M., Heinrich, I., Schollän, K., Balanzategui, D., Schürheck, L., 2022. Stable Isotope Signatures of Wood, its Constituents and Methods of Cellulose Extraction, in: Siegwolf, R.T.W., Brooks, J.R., Roden, J., Saurer, M. (Eds.), Stable Isotopes in Tree Rings: Inferring Physiological, Climatic and Environmental Responses. Springer International Publishing, Cham, pp. 135–190. https://doi.org/10.1007/978-3-030-92698-4_5

Leppä, K., Tang, Y., Ogée, J., Launiainen, S., Kahmen, A., Kolari, P., Sahlstedt, E., Saurer, M., Schiestl-Aalto, P., Rinne-Garmston, K.T., 2022. Explicitly accounting for needle sugar pool size crucial for predicting intra-seasonal dynamics of needle carbohydrates δ18O and δ13C. New Phytologist 236, 2044–2060. https://doi.org/10.1111/nph.18227

Ogée, J., Barbour, M.M., Wingate, L., Bert, D., Bosc, A., Stievenard, M., Lambrot, C., Pierre, M., Bariac, T., Loustau, D., Dewar, R.C., 2009. A single-substrate model to interpret intra-annual stable isotope signals in tree-ring cellulose. Plant, Cell & Environment 32, 1071–1090. https://doi.org/10.1111/j.1365-3040.2009.01989.x

Schleser, G.H., Anhuf, D., Helle, G., Vos, H., 2015. A remarkable relationship of the stable carbon isotopic compositions of wood and cellulose in tree-rings of the tropical species *Cariniana micrantha* (Ducke) from Brazil. Chemical Geology 401, 59–66. https://doi.org/10.1016/j.chemgeo.2015.02.014

Tang, Y., Sahlstedt, E., Young, G., Schiestl-Aalto, P., Saurer, M., Kolari, P., Jyske, T., Bäck, J., Rinne-Garmston, K.T., 2023. Estimating intraseasonal intrinsic water-use efficiency from high-resolution tree-ring δ13C data in boreal Scots pine forests. New Phytologist 237, 1606–1619. https://doi.org/10.1111/nph.18649

Walcroft, A.S., Silvester, W.B., Whitehead, D., Kelliher, F.M., 1997. Seasonal Changes in Stable Carbon Isotope Ratios within Annual Rings of Pinus radiata Reflect Environmental Regulation of Growth Processes. Functional Plant Biol. 24, 57–68. https://doi.org/10.1071/pp96025

Yoshimura, K., Frankenberg, C., Lee, J., Kanamitsu, M., Worden, J., Röckmann, T., 2011. Comparison of an isotopic atmospheric general circulation model with new quasi-global satellite measurements of water vapor isotopologues. Journal of Geophysical Research: Atmospheres 116. https://doi.org/10.1029/2011JD016035

Yoshimura, K., Kanamitsu, M., Noone, D., Oki, T., 2008. Historical isotope simulation using Reanalysis atmospheric data. Journal of Geophysical Research: Atmospheres 113. https://doi.org/10.1029/2008JD010074
